# Comparison of Spectrum Estimation Methods for the Accurate Evaluation of Sea State Parameters †

**DOI:** 10.3390/s20051416

**Published:** 2020-03-05

**Authors:** Giovanni Battista Rossi, Francesco Crenna, Vincenzo Piscopo, Antonio Scamardella

**Affiliations:** 1 Department of Mechanical, Energy, Management and Transportation Engineering, University of Genova, Via Opera Pia 15A, 16145 Genova, Italy; crenna@dimec.unige.it; 2 Department of Science and Technology, University of Naples “Parthenope”, Centro Direzionale Isola C4, 80143 Naples, Italy; vincenzo.piscopo@uniparthenope.it (V.P.); antonio.scamardella@uniparthenope.it (A.S.)

**Keywords:** sea state parameters, welch method, Thomson method, parametric estimation method, Sea state reconstruction iterative procedure, non-linear least square method

## Abstract

The monitoring of sea state conditions, either for weather forecasting or ship seakeeping analysis, requires the reliable assessment of the sea spectra encountered by the ship, either as a final result or intermediate step for the measurement of the relevant wave-motion parameters. In current analyses, different spectrum estimation methods, namely the Welch, Thomson and ARMA models, have been applied and compared based on a set of random wave signals, with different durations, representative of several sea state conditions. Subsequently, two sea spectrum reconstruction techniques were described and applied in order to detect the main sea state parameters, namely the significant wave height, the mean wave period and the spectrum peak enhancement factor. The performances of both spectral analysis and sea state reconstruction methods are discussed in order to provide some preliminary guidelines for practical application purposes. In this respect, based on current results, the Welch and Thomson methods seem to be the most promising techniques, combined with the nonlinear least-square reconstruction technique.

## 1. Introduction

Nowadays ships are equipped with a variety of sensors useful to obtain information about relevant motions and accelerations in a seaway and to increase, by means of proper active weather routing techniques, both the level of onboard comfort experienced by passengers [1] and crew safety during routine working operations. In this respect, the correct estimate of ship motion spectra is a basic issue to provide reliable information to the active weather routing decision system and increase the onboard comfort level and the safety of ship and navigation.

Before assessing the main parameters of the ship motion spectra in a seaway, the basic problems of sea spectrum reconstruction from wave time history needs to be investigated, concerning: (i) the proper selection of the most reliable technique that allows for efficiently reconstructing the wave spectrum; (ii) the minimum duration of the wave time record, required to obtain a robust estimate of the wave spectrum parameters; (iii) the amplitude of the window embodied in the Short-Time Fourier transform (STFT) when evolving sea states need to be investigated. These basic issues are essential for the optimal fitting of wave spectra to measured sea states, as widely discussed, e.g., by Mansard and Funke [2].

Therefore, a study has been undertaken in order to compare the performances of some key spectrum estimation methods, whose preliminary results were anticipated in the 2019 International Workshop on Metrology for the Sea IMEKO TC 19, held in Genoa, Italy, in 2019 [3]. In this study, signals describing four significant sea states were generated, according to the Joint North Sea Wave Project (JONSWAP) spectrum model, of both long (one hour) and short (ten minutes) durations. They were processed through three different spectrum estimators, both non-parametrical (Welch’s and Thomson’s methods) and parametrical—the latter based on an Auto-Regressive Moving Average (ARMA) model—and results were compared with respect to their capability of reconstructing the theoretical spectrum-model that originated the signals.

Then, the estimation of three main sea state parameters—namely the wave peak frequency, the peak enhancement factor and the significant wave—from such spectra was considered. In terms of the estimation of the wave peak frequency, different peak frequency estimators are available in literature [2]. The peak enhancement factor is generally assessed using a bivariate Rayleigh probability density function, while the significant wave height is generally estimated using the truncated zero-order spectral moment [4]. All these methods have been considered and tested and, in addition to them, the estimation of such parameters based on a non-linear fitting of the spectral model to the estimated spectra has been applied and results have been compared. Guidelines for the application of both spectrum estimation and parameter estimation methods are finally provided.

## 2. Monitoring of Sea State Parameters

Sea state parameters are key factors for assuring the safety of a ship and navigation, and relevant monitoring is a basic issue in monitoring the ship’s motions in a seaway and, eventually, selecting the optimal route based on adaptive weather routing criteria. Besides, it is well-known that real sea state conditions are characterized by random waves; generally described by Pierson–Moskowitz (PM) or JONSWAP spectra—generally applied for fully and partly developed seas. Fully developed seas occur when the wind blows for a long period over a wide area of sufficient fetch length, while partly developed seas occur in fetch-limited conditions. Hence, in current analyses, the JONSWAP spectrum $S_{J}$ is applied according to the following equation format [5]:

(1) $$S_{J}\left(f\right)\;=\;A_{\gamma }S_{PM}\left(f\right)\gamma^{exp\left(-0.5{\left(\frac{f-f_{p}}{\sigma f_{p}}\right)}^{2}\right)}$$

having denoted by: f the wave frequency, $A_{\gamma }\;=\;1-0.287ln\gamma$ the normalizing factor, σ the spectral width parameter, equal to 0.07 if $f\leq f_{p}$ and 0.09 otherwise, $f_{p}$ the peak frequency, γ the peak enhancement factor and $S_{PM}$ the Pierson–Moskowitz spectrum:

(2) $$S_{PM}\left(f\right)=\frac{5}{16}H_{s}^{2}f_{p}^{4}f^{-5}exp\left(-\frac{5}{4}{\left(\frac{f}{f_{p}}\right)}^{-4}\right)$$

Hence, the PM spectrum for fully developed seas (γ = 1) is a special case of the JONSWAP one.

## 3. Spectrum Estimation Methods

### 3.1. Outline of Some of the Main Spectrum Estimation Methods

Spectrum measurement is a key tool for the monitoring of dynamic phenomena. To perform such a measurement, signals have firstly to be acquired, through a measurement system with adequate dynamic characteristics (frequency response), and processed in order to obtain an estimate of the spectrum. Several estimation methods have been proposed since the appearance of the Fast Fourier Transform (FFT), in the late 1960s, which made their implementation as digital procedures possible. They may be parsed in two main groups—namely non parametrical and parametrical [6,7]. The seminal idea under the first group was the “periodogram”, that is the square of the Fourier Transform (FT) of the series of observations, normalized in respect of the observation duration, $T_{0}$. The periodogram, first proposed by Schuster [8] as a way for identifying hidden periodicities in a highly noisy signal, constitutes a rough estimate of the power spectral density (PSD), since it is both biased, for a limited observation time, and it shows a high variance, which does not decrease by increasing the observation time. In fact, a longer observation time allows better spectral resolution, since the frequency spacing in the measured spectrum is $\Delta f\;=\;T_{0}^{-1}$, but the variance remains the same. The basic periodogram can, and should, be improved to reduce bias, which is accomplished typically by tapering or pre-whitening procedures, and to reduce variance, by averaging or smoothing. An important method that moves along these lines, is the one proposed by Welch in 1967 [9], which has been considered in the present work.

Another important method was proposed by Thomson [10], still in the area of the non-parametrical approach. The basic idea was to taper the data with different tapers to highlight different features of the signal, hence the denomination of *multi-taper method* (MTM).

An approach alternative to the non-parametrical is called parametrical and has been developed since the 1970s. It basically consists of considering parametrical models of the observed time series, such as the *auto-regressive moving-average* (ARMA) model, and obtaining estimates for the parameters involved. Once such estimates are available, the PSD of the signal may be approximated by that of the output of the model when driven by a white noise input. Methods differ in the type of model (AR, MA, or ARMA) and in the way the parameters are estimated.

### 3.2. Welch Method

The method consists in parsing the data record, corresponding to an (overall) observation duration T, in smaller segments of duration $T_{0}$, with partial overlap, typically from 20% to 50%. Each segment is pre-treated by tapering with a smooth window, to reduce the bias due to spectral leakage, then the (modified) periodogram is calculated for each of them. The spectrum is obtained by averaging over such periodograms. In this way, bias is reduced by tapering and variance is reduced by averaging.

Let us then denote the series of measurements by $x_{i}=x\left(i\Delta t\right)$, where Δt is the sampling interval, and i=1,…N, with T=NΔt, and $T_{0}=N_{0}\Delta t$. Let $w_{1},\ldots ,w_{N_{0}}$ be a data taper, then the modified periodogram for the l-th segment is:

(3) $$\;{\hat{S}}_{l}\left(f\right)=\Delta t{\left|\overset{N_{0}}{\underset{i=1}{\sum }}w_{i}x_{i+l-1}e^{-j2\pi fi\Delta t}\right|}^{2}$$

where j is the imaginary unit. The spectral estimator is then:

(4) $$\hat{S}\left(f\right)=\frac{1}{n}\overset{n-1}{\underset{k=0}{\sum }}{\hat{S}}_{km+1}\left(f\right)$$

where n is the number of segments and m is an integer-valued shift factor, satisfying $0<m\leq N_{0}$ and $m\left(n-1\right)=N-N_{0}$.

Let us briefly discuss the application of the method and the criteria for choosing the involved parameters. Basically, two main “metrological” characteristics of the method must be considered, namely the effective bandwidth and the variance (or, equivalently, the standard deviation). It should be noted that the bandwidth of a spectral estimator is a measure of the minimum separation in frequency between approximately uncorrelated spectral estimates [7]. Therefore, the wider such bandwidth is, the worse spectral resolution is. Let us then briefly discuss the choice of the main parameters of the method. The degree of overlap is strictly related the kind of taper adopted. Basically, the smoother the taper is, the higher the degree of overlap can be adopted, which allows us to increase the number of segments and to reduce the variance. On the other hand, the smoother the window is, the larger its bandwidth is and, consequently, the worse its spectral resolution results. Welch suggested adopting a 50% overlap, and to apply a cosine (Hanning) window: we consider this a very good compromise and thus we have adopted it in the present study. Concerning the effective bandwidth, for Welch’s method it can be expressed as $\Delta f_{e}=\alpha_{w}T_{0}^{-1}$ where $\alpha_{w}$ is a factor that depends upon the kind of the selected taper and on the way bandwidth is defined. In the case of the Hanning window and considering a half-power bandwidth, we obtain $\alpha_{w}=1.44$ [6]. Concerning the variance of the estimator, with a 50% overlap, a relative standard uncertainty (standard deviation) $u_{S}\left(f\right)/S\left(f\right)=\sqrt{\left(11/18\right)N_{0}N^{-1}}$ can be assumed, where S(f) is the PSD and $u_{S}\left(f\right)$ is the absolute standard uncertainty [10]. These results are of great metrological import, since they allow us to keep the quality of the result under control. Once the record duration T is fixed and the kind of taper and the degree of overlap have been decided, the duration of the observation window, $T_{0}$, remains the only design parameter to be optimised. Such optimization can be done using a trial-and-error approach, with a trade-off between the need to have a good spectral resolution, which demands for a large $T_{0}$, and a small variance, which requires a small $T_{0}.$ It is important to note that in each trial the associated effective bandwidth and variance can be computed thanks to the above formulae, which are valid only for the Hanning window with a 50% overlap. Details of the choice made in this study will be given in Section 5.

### 3.3. Thomson Method

This method generalizes the tapering issue by adopting multiple orthogonal tapers. The aim is to recover the information that may be lost when using a single taper. The estimator is the average of K direct spectral estimators, each acting on the whole data record (rather than on a signal segment, as happens in Welch method) and applying a different taper. Each (partial) estimator is defined by:

(5) $${\hat{S}}_{k}\left(f\right)=\Delta t{\left|\overset{N}{\underset{i=1}{\sum }}h_{i,k}x_{i+l-1}e^{-j2\pi fi\Delta t}\right|}^{2}$$

where $h_{i,k}$ is the kth data taper, usually chosen as the kth discrete prolate spheroidal sequence with parameter W, where 2W is the normalized bandwidth of the tapers, i.e., the bandwidth for Δt=1 s. The final estimator is thus:

(6) $$\hat{S}\left(f\right)=\frac{1}{K}\overset{K-1}{\underset{k=0}{\sum }}{\hat{S}}_{k}\left(f\right)$$

where K is typically chosen to be equal to 2NW−1. The metrological characteristics of the procedure can be kept under control by assuming an effective bandwidth $\Delta f_{e}=2W/\Delta t$ (Hz) and considering that the estimator is approximately equal in distribution to $S\left(f\right)\chi_{2K}^{2}/2K$, which yields a relative standard uncertainty equal to $K^{-\frac{1}{2}}$. Therefore, in respect to Welch method, there is here much less arbitrariness, since, for a fixed observation time, T, the only parameter to be chosen is the half-bandwidth W, which influences both spectral resolution and relative standard uncertainty. Detail on the choices made in this study will be given in Section 5.2

### 3.4. Parametric Estimation Methods (ARMA)

An alternative class of methods, called parametrical, consider discrete parametrical time models of the series of observations and look for estimators of the involved parameters. Once that estimates are obtained for them, the spectrum can be calculated as a function of such parameters. For example, in the special case of (zero-mean) auto-regressive moving-average (ARMA) models, the series of measurements $x_{i}$ are modelled as:

(7) $$x_{i}=-a_{1}x_{i-1}\ldots -a_{p}x_{i-p}+\epsilon_{i}+b_{1}\varepsilon_{i-1}\ldots +b_{q}\varepsilon_{i-q}$$

where $\varepsilon_{i}$ is a white noise process with zero mean and variance $\sigma^{2}$, and $a_{k}$ and $b_{k}$ are the parameters of the model. Their estimation involves solving a non-linear optimization problem, for which some methods are available, none of which, to the authors’ knowledge, prevails over the others. Once estimated, ${\hat{a}}_{k}$ and ${\hat{b}}_{k}$, are available and $\sigma^{2}$ has also been estimated as the mean squared error (MSE) of the optimization process, the spectrum can be calculated as follows:

(8) $$S\left(f\right)={\hat{\sigma }}^{2}\Delta t{\left|\frac{1+\sum_{k=1}^{q}{\hat{b}}_{k}e^{-j2\pi fk\Delta t}}{1+\sum_{k=1}^{p}{\hat{a}}_{k}e^{-j2\pi fk\Delta t}}\right|}^{2}$$

Historically, parametrical methods were developed mainly to overcome limitations in achievable spectral resolution that are inherent in the non-parametrical approach, especially for short records. Here, the main design parameter is the choice of the model order n, which is a somewhat critical issue. In fact, the ARMA model basically refers to the response of a linear system to a broad-band, flat-spectrum excitation, which may be an acceptable assumption, e.g., in vibration measurement, in many cases. If the system under investigation may be modelled as linear, one could consider the order of the assumed model. Yet even in this favorable case, there may be problems due, for example, to minor modes in the real system not predicted by the model, of by a not flat spectrum excitation—both circumstances requiring additional degrees of freedom for being properly described, which implies a higher model order. Furthermore, when the above assumption is not justified, as in the case of the JONSWAP spectrum, the ARMA model can only be regarded as a flexible mathematical device capable of approximating a wide range of situations. Therefore, criteria have been proposed for helping model order selection, based on purely statistical/informational criteria. One the most popular in Akaike’s final prediction error (FPE) criterion [6]. In this study, we have searched, for each test case, the best order both by comparing the estimated and the theoretical spectrum through visual inspection, and by considering the FPE also. Results will be discussed in Section 5.2.

## 4. Assessment of Sea State Parameters

### 4.1. Iterative Procedure

**Figure 1:**
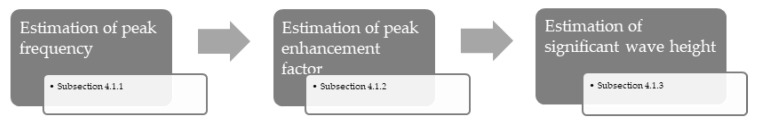
Flow chart of the iterative procedure.

The iterative procedure proposed by Mansard and Funke [2,4] is mainly based on three steps, devoted to the assessment of the spectrum peak frequency, peak enhancement factor and significant wave height, respectively, according to the flow chart shown in Figure 1. In order to assess the peak frequency at the first step of the iterative procedure, a tentative value equal to 1.385 is assumed for the peak enhancement factor, as detailed in the following.

#### 4.1.1. Estimation of the Peak Frequency

The assessment of the spectrum peak frequency $f_{p}$ can be performed based on different algorithms, as discussed by Mansard and Funke [2,4]. The detection of the peak period based on the largest value of the reconstructed wave spectrum could lead to a large variability of the peak frequency and should be avoided for practical purposes. In this respect, Mansard and Funke concluded that the best estimate of the peak frequency can be obtained by applying the method proposed by Read [11], and mainly based on the 5th order spectral moment of the reconstructed wave spectrum. Furthermore, they introduced in the formula provided by Read a bias corrective factor $C_{f}$ depending on the spectrum peak enhancement factor γ, according to the following equation:

(9) $$f_{p}=\frac{1}{C_{f}}\int_{0}^{\infty }fS^{5}\left(f\right)df/\int_{0}^{\infty }S^{5}\left(f\right)df$$

where $C_{f}$ is defined as follows:

(10) $$C_{f}=1.005+1/\left[50.746{\left(\gamma -0.2397\right)}^{2}\right]$$

The corrective factor provided by Equation (10) can be determined once the peak enhancement factor is known, which implies that an iterative procedure is required. Hence, Mansard and Funke [2,4] suggest assuming it equal to 1.02, namely γ=1.385, at the first step of the iterative procedure to compute the peak enhancement factor γ at the subsequent step.

#### 4.1.2. Estimation of the Peak Enhancement Factor

The peak enhancement factor can be determined after assessing the shape parameter $k_{f}$ of the bivariate Rayleigh distribution provided by Battjes and van Vledder [12]:

(11) $$k_{f}^{2}m_{0}^{2}={\left[\int_{0.5f_{p}}^{2.5f_{p}}S\left(f\right)cos\left(2\pi f\tau \right)df\right]}^{2}+{\left[\int_{0.5f_{p}}^{2.5f_{p}}S\left(f\right)sin\left(2\pi f\tau \right)df\right]}^{2}$$

where $\tau =\sqrt{m_{0}/m_{2}}$ depends on the zeroth and second order spectral moments of the reconstructed wave spectrum, determined based on a lower and upper bounds of the wave frequency equal to $0.5f_{p}$ and $2.5f_{p}$, respectively. After assessing the shape parameter $k_{f}$, the tentative peak enhancement factor $\gamma_{0}$ is assessed by the following equation:

(12) $$\gamma_{0}=\{\begin{array}{l} 50.69-404.97k_{f}+1211.2k_{f}^{2}-1599.6k_{f}^{3}+817.26k_{f}^{4}\;if\;k_{f}\geq 0.4\;\\ 1\;otherwise \end{array}$$

After determining $\gamma_{0}$, the peak enhancement factor is determined by the following formula provided by Mansard and Funke [2,4]:

(13) $$\gamma =\{\begin{array}{l} -0.835+1.797\gamma_{0}-0.2011\gamma_{0}^{2}\;if\;\gamma_{0}<0.4\;\\ \gamma_{0}-0.10\;otherwise \end{array}$$

**Figure 2:**
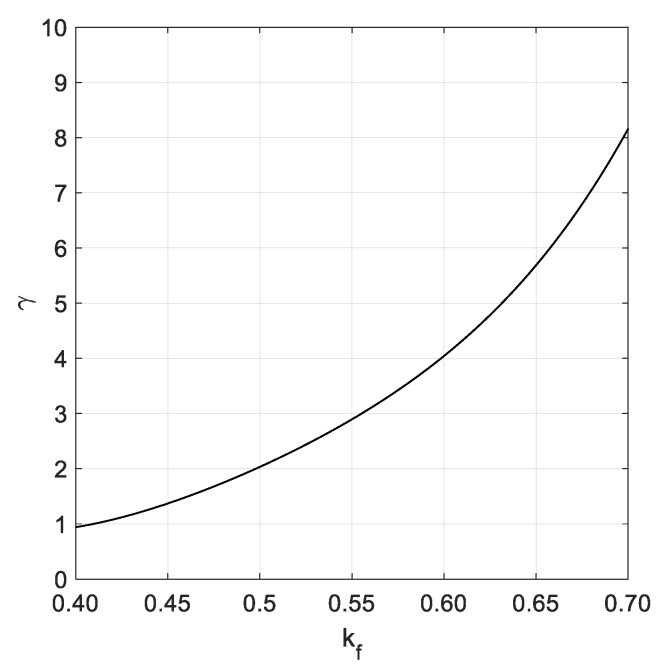
Dependence of γ upon $k_{f}$.

This corrective term was provided to reduce the bias in the assessment of γ, based on random generation of sea state elevations starting from theoretical JONSWAP spectra. Figure 2 provides the plot of the peak enhancement factor γ versus the shape parameter $k_{f}$ of the bivariate Rayleigh distribution that generally ranges from 0.4 up to 0.7 for practical purposes. Hence, after assuming in Equation (10) a tentative value of the peak enhancement factor equal to 1.385, the peak frequency $f_{p}$ is determined by Equation (9) and the γ value is updated by Equation (13). The procedure is iteratively performed until the relative variation of the peak enhancement factor between two subsequent steps is lower than a given threshold that can be assumed equal to 1% for practical purposes.

#### 4.1.3. Estimation of the Significant Wave Height

The significant wave height $H_{s}$ is determined by the following formula:

(14) $$H_{s}=4\sqrt{m_{0,c}}$$

where $m_{0,c}$ is the corrected zeroth spectral moment [2,4]:

(15) $$m_{0,c}={\left[1.0015+\frac{1}{19.9178\left(\gamma +2.6937\right)}\right]}^{2}m_{0}$$

The term provided in Equation (15) allows removing the bias in the significant wave height assessment.

### 4.2. Nonlinear Least-Square Method

The nonlinear least-square method (NLSM), proposed by Rossi et al. [3], is mainly based on the preliminary assessment of the significant wave height based on zeroth order spectral moment of the reconstructed wave spectrum, according to the following equation:

(16) $$H_{s}=4\sqrt{m_{0}}$$

The spectrum peak frequency and enhancement factor are subsequently detected by curve fitting of the theoretical JONSWAP spectrum based on the least-square method, which is based on the iterative trust-region-reflective algorithm, according to the interior-reflective Newton method [13]. Each iteration involves the approximate solution of a large linear system using the method of preconditioned conjugate gradients.

## 5. Numerical Study of Selected Test Cases

### 5.1. Selection of Test Cases and Random Wave Generation

In the numerical study the test cases reported in Table 1 have been considered, as they cover a wide range of sea state conditions, including both fully (γ=1) and partly (γ>1) developed sea states and corresponding to grades 3, 4, 5 and 6 of the Douglas (DG) Scale that allows connecting the significant wave height with roughness of sea for navigation. In Table 1 $H_{s}$ denotes the significant wave height, while $T_{m}$ ($T_{p}$) is the mean (peak) wave period.

**Figure 3:**
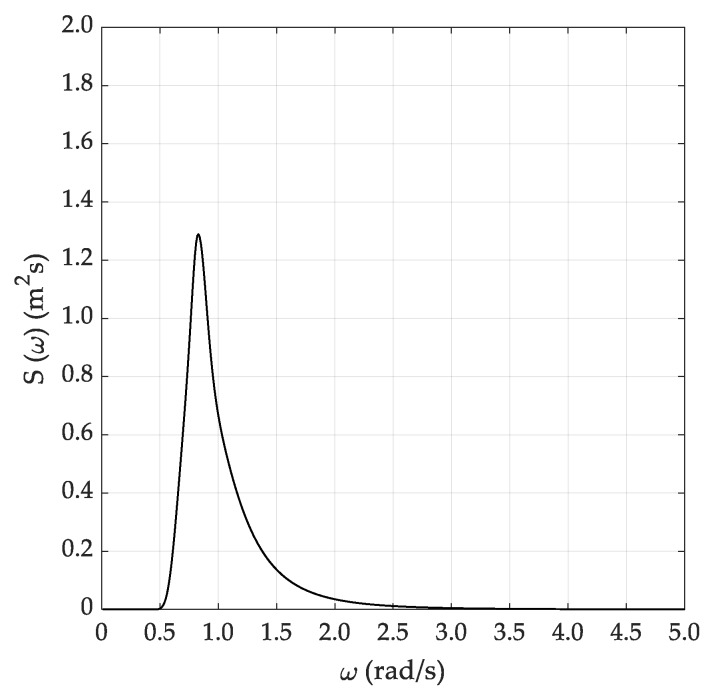
Input JONSWAP spectrum (DG5).

**Figure 4:**
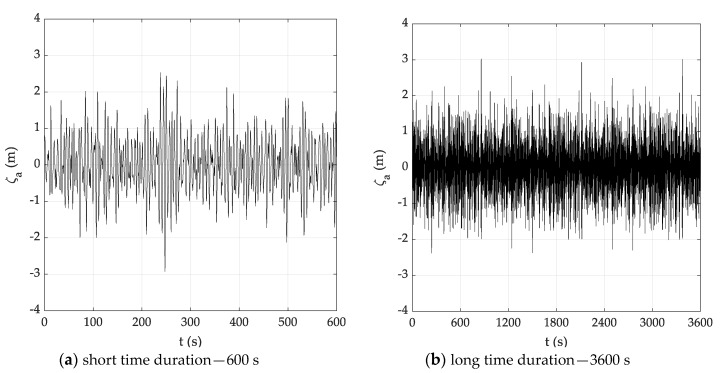
Random wave time histories (DG5).

For each sea state condition, two random wave time histories have been generated based on 600 (short) and 3600 s (long) time durations, starting from the theoretical JONSWAP spectra. Hence, Figure 3 reports the input JONSWAP spectrum for the DG5 sea state condition, while Figure 4a,b report the short and long random wave time histories. In this respect, it must be pointed out that the wave time history was determined based on the following equation:

(17) $$\varsigma_{a}\left(t\right)=\overset{n}{\underset{i=1}{\sum }}\sqrt{2S\left(f_{i}\right)df_{i}}cos\left[2\pi f_{i}t+\varphi_{i}\right]$$

After partitioning the theoretical input spectrum into a discrete set of components. In Equation (17) $\varsigma_{a}$ denotes the wave amplitude, while $f_{i}$ is the *i*-th wave frequency component, with uniform random phase $\varphi_{i}$ in the interval [0,2π].

### 5.2. Spectral Analysis

**Figure 5:**
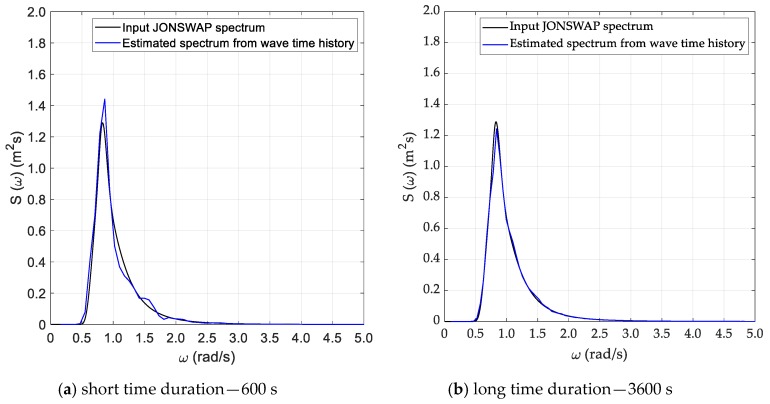
Estimated sea spectrum—Welch method (DG5).

**Figure 6:**
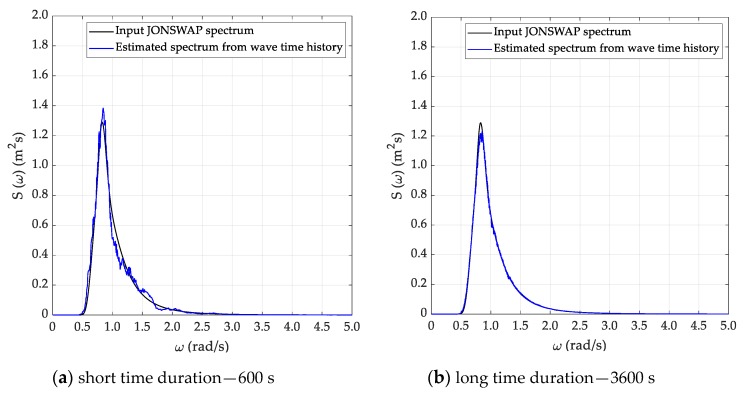
Estimated sea spectrum—Thomson method (DG5) 7.

**Figure 7:**
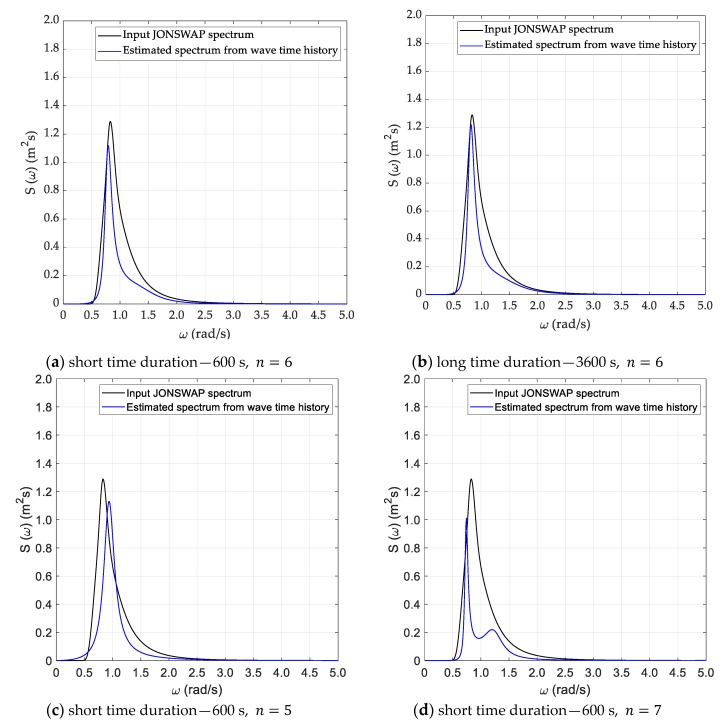
Estimated sea spectrum—ARMA method (DG5).

Spectral analysis of the time series generated as specified in the previous section was performed according to the three methods described in Section 3, namely Welch, Thomson and ARMA. Results for the DG5 sea state are provided, in graphical form, in Figure 5, Figure 6 and Figure 7, whilst results from all test cases with reference to the recovered sea state parameters are provided, in tabular form, in Table 2, Table 3, Table 4 and Table 5, in Section 5.3, where they are also discussed.

As anticipated in Section 3.2, the Welch method was applied with the classical Hanning (cosine) data window and with a 50% superposition of adjacent segments. For the long (T=3600 s) time series, a segment duration $T_{0}=120\;s$ was adopted, which corresponds to an effective bandwidth $\Delta f_{e}=0.012\;\mathrm{Hz}$ and to a relative standard uncertainty (standard deviation) $u_{S}\left(f\right)/S\left(f\right)=0.14$. In the case of short records, a higher (worse) estimator’s bandwidth was accepted, to limit the uncertainty of the estimate. Therefore $T_{0}=80\;s$ was adopted, which implies an effective bandwidth $\Delta f_{e}=0.018\;\mathrm{Hz}$ and yields a relative standard uncertainty (standard deviation) $u_{S}\left(f\right)/S\left(f\right)=0.29$. These choices were based on the previous experience gained in the preliminary study reported in Ref [3] and its validity was checked and confirmed in the present investigation. Results from this method for sea state DG5, are presented in Figure 4. The results are quite good, especially for the long record, whilst for the shorter one the trade-off between resolution and statistical variability is more critical, as the quoted figures clearly show. In fact, this analysis is quite demanding, since in addition to a limited variance a good spectral resolution is required, to properly represent the narrow peak of the spectrum. In fact, differences between the theoretical and the estimated spectrum are predicted even in a conservative way by the relative uncertainty figures calculated for the method.

In the application of Thomson method, a similar trade-off was made for the choice of the leading parameter for this analysis, that is the normalized half bandwidth of the taper W. In the case of the long duration signal, W=0.0042 was assumed, which corresponds to an effective bandwidth $\Delta f_{e}=0.017\;\mathrm{Hz}$ [14] and to relative standard uncertainty $u_{S}\left(f\right)/S\left(f\right)=0.13$ [15]. In the case of the short duration signal, W=0.0063 was taken instead, yielding $\Delta f_{e}=0.025\;\mathrm{Hz}$ and $u_{S}\left(f\right)/S\left(f\right)=0.27$ respectively. Results from this analysis are shown in Figure 6. In spite of their quite “noisy” appearance, which is somewhat typical of this method, originally conceived for dealing with short records, they are reliable and comparable, if not even superior, to the ones provided by the Welch method, as concerns their capability of restoring the shape of the main peak of the spectrum, which we consider the most important goal, in this context. A deeper comparison will be possible in reference to the recovery of sea state parameters, to be discussed in Section 5.3.

Concerning parametrical spectrum estimation, a preliminary investigation was reported in [3] where Autoregressive (AR) models were considered [16], with two well established estimation methods, namely Burg’s algorithm and the covariance approach [6]. Results were not fully satisfactory since for low model orders (2–6) some bias in the localization of the spectrum peak was experienced, whilst when increasing the order spurious artefact peaks emerged. Therefore, in the present study ARMA models were considered instead, since they can usually provide comparable or superior approximation in respect to AR models, with lower model orders [17]. In fact, the results were better, since peak localization, with the proper model order, can now be achieved, although the resulting peak shape is still not fully satisfactory. As mentioned in Section 3.4, the search of the best model order was done both by comparing the estimated spectrum with the reference one, for various, progressively increasing orders, and by calculating the corresponding Akaike’s FPE. A general pattern appeared: for low orders a poor reconstruction of the main peak resulted; this improved to some extent but proceeding further spurious peaks appeared. This behavior is similar to what happened with the AR estimator [3], but the best results here were much better than in the AR approach. In most cases order 6 led to the best results, yet in some case orders up to 10 had to be assumed. The FPE was also computed, which provided results in agreement with the visual inspection in 40% of cases. Results for this approach as applied to signal DG5 are shown in Figure 7a for a short time duration, and Figure 7b, for a long time one. In both cases model order n=6 yielded the best results.

Furthermore, in Figure 7c,d, results for the short signal for n=5 and for n=7, respectively, are shown. In this case, model order 6 was selected. It can be noted that for the lower order, there is an evident mismatch in the localization of the main peak, whilst with the higher order, a spurious second peak appears. In this case, for example, the FPE criterion rated order n=7 better than order n=6, which is clearly not the case.

### 5.3. Sea State Reconstruction

Sea state reconstruction was performed based on the sea spectrum analysis carried out in Section 5.2. Particularly, the significant wave height *H_s_*, the mean wave period *T_m_*, and the peak enhancement factor *γ* were determined by the iterative and NLSM procedures, outlined in Section 4.1 and Section 4.2. The reconstructed parameters, corresponding to the DG3 sea state, are reported in Table 2 for both the short and long time durations, corresponding to 600 and 3600 s, respectively. Based on current results, it can be seen that:

(i) The Welch and Thomson methods allow reconstructing the sea state parameters with relative errors lower than 3% for the significant wave height and mean period and 8% for the peak enhancement factor.
(ii) Higher errors arise if the ARMA method is applied, especially for the assessment of the spectrum peak enhancement factor, with absolute percentage errors up to 50%.
(iii) The accuracy of the methods generally increases with the time duration, even if the reliability of reconstructed parameters is sufficiently accurate for practical purposes, even if the short time history, corresponding to 600 s, is embodied in the spectral analysis. Nevertheless, this trend is not always clear, provided that a certain dependence on both the spectrum estimation method and reconstruction technique is also recognized.

**Figure 8:**
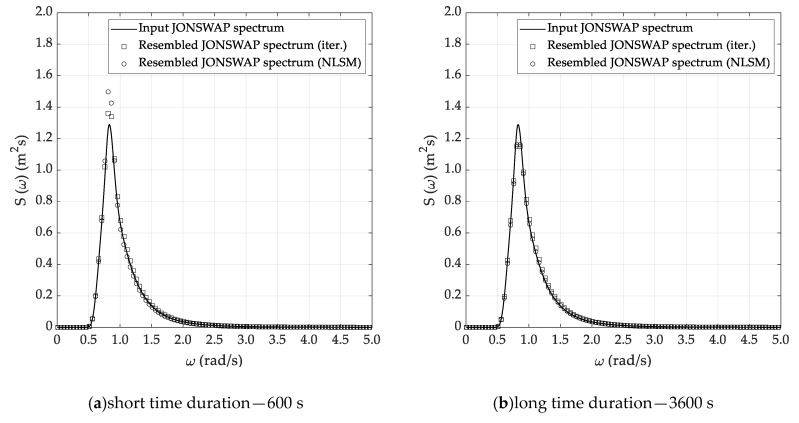
Reconstructed sea spectrum—Welch method (DG5).

**Figure 9:**
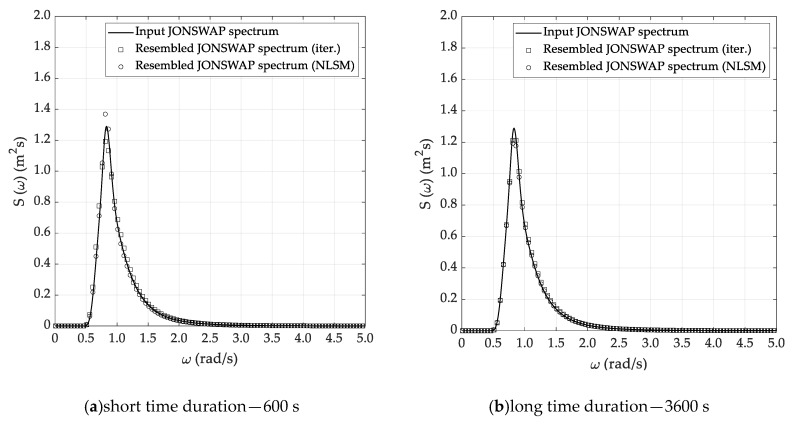
Reconstructed sea spectrum—Thomson method (DG5).

**Figure 10:**
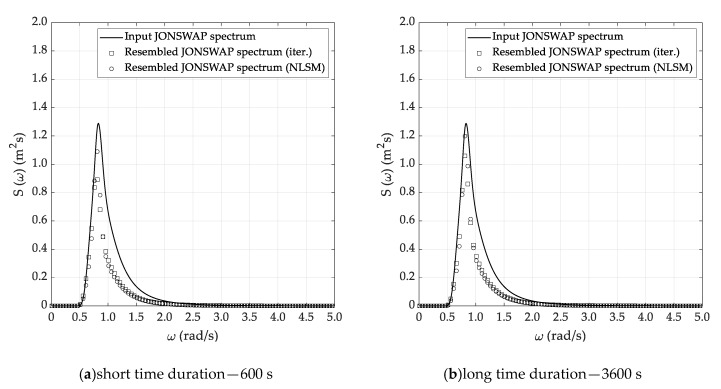
Reconstructed sea spectrum—ARMA method (DG5).

Almost similar outcomes can be stressed by the analysis of the results reported in Table 3, Table 4 and Table 5, corresponding to the DG4, DG5 and DG6 sea state conditions. In fact, in all cases it is confirmed that the Welch and Thomson methods allow the efficient reconstruction of the sea state parameters, while some issues arise when using the ARMA method. Concerning the selection of the most suitable sea state reconstruction technique, no substantial differences arise between the iterative and NLSM methods. In fact, the percentage errors are comparable—even if it seems that the latter technique is slightly more accurate than the former and thus preferable, according to the results of the simulations. Starting from the results listed in Table 2, Table 3, Table 4 and Table 5, Figure 8, Figure 9 and Figure 10 report a comparative analysis between the theoretical and reconstructed JOSNWAP spectra for the DG5 sea state condition, with reference to both the short and long time durations. The reference spectrum is plotted in a continuous line, while the square and circle lines refer to the sea spectra reconstructed by the iterative and NLSM methods, respectively.

The current results confirm the main outcomes already stressed by the analysis of the results reported in Table 2, Table 3, Table 4 and Table 5. In fact, it is not only confirmed that the Welch and Thomson methods allow the efficient reconstruction of the theoretical sea spectrum—it is also verified that the ARMA method fails to adequately predict the spectrum peak enhancement factor. Obviously, the reconstructed sea spectrum, corresponding to the long time duration, matches the theoretical JONSWAP spectrum slightly better, even if the results obtained based on the short time duration are not fully reliable for practical purposes.

## 6. Conclusions

The paper focused on the application of three spectral analysis techniques, combined with two sea state reconstruction methods, with the main aim of deriving the sea state parameters, namely the significant wave height, the mean wave period and the spectrum peak enhancement factor, starting from a random wave time history. In particular, two random sea surface elevations, corresponding to 600 and 3600 s, were generated, based on four theoretical JONSWAP spectra, representative of the DG3, DG4, DG5 and DG6 sea state conditions.

The Welch, Thomson and ARMA methods were applied to derive the sea spectra from the wave time histories, representative of the selected sea state conditions. In this experiment, it was possible to identify optimal values for the key analysis parameters, that may constitute a useful indication for the practical application of these methods. Subsequently, the sea state parameters of the reconstructed JONSWAP spectra were determined by the iterative and NLSM methods, in order to detect the most suitable combination of spectral analysis and sea state reconstruction techniques. Based on current results, the Welch and Thomson methods seem to be the most promising techniques, combined with the NLSM method, as they allow us to efficiently reconstruct the sea state parameters, even in the short-time duration case. Contrastingly, it was verified that the ARMA method fails to adequately predict the spectrum peak enhancement factor. Current outcomes are promising for further research activities, devoted to investigating the effectiveness of the proposed techniques with reference to real sea state conditions, often characterized by double-peaked wave spectra, obtained by combining wind sea and swell waves coming from different directions. This topic will be the subject of future work.

## Figures and Tables

**Table 1:** Selected sea state conditions.

DG	Sea State Condition	Hs	Tm	Tp	γ
		[m]	[s]	[s]	[—]
3	Slight	1.00	4.00	4.82	3.00
4	Moderate	2.00	5.00	6.11	2.50
5	Rough	3.00	6.00	7.59	1.50
6	Very rough	5.00	9.00	11.64	1.00

**Table 2:** Reconstructed sea state parameters (DG3).

(a) Short Time Duration—600 s.				(b) Long Time Duration—3600 s.			
Method/Difference	Hs	Tm	γ	Method/Difference	Hs	Tm	γ
	[m]	[s]	[—]		[m]	[s]	[—]
*Input data*				*Input*			
JONSWAP	1.000	4.000	3.000	JONSWAP	1.000	4.000	3.000
*Welch method*				*Welch method*			
Iterative	0.995	4.110	2.927	Iterative	1.012	3.983	2.915
NLSM	0.985	4.055	2.794	NLSM	1.002	3.975	2.759
Δ_iter._ (%)	−0.495	2.743	−2.426	Δ_iter._ (%)	1.236	−0.421	−2.849
Δ_NLSM_ (%)	−1.523	1.385	−6.883	Δ_NLSM_ (%)	0.189	−0.637	−8.044
*Thomson method*				*Thomson method*			
Iterative	0.999	4.088	3.140	Iterative	1.011	3.994	2.999
NLSM	0.989	4.056	2.979	NLSM	1.001	3.988	2.904
Δ_iter._ (%)	−0.058	2.212	4.665	Δ_iter._ (%)	1.092	−0.143	−0.046
Δ_NLSM_ (%)	−1.058	1.412	−0.702	Δ_NLSM_ (%)	0.059	−0.289	−3.188
*ARMA method*				*ARMA method*			
Iterative	1.024	3.979	4.000	Iterative	0.783	3.788	1.517
NLSM	1.015	3.920	3.084	NLSM	0.773	3.995	4.303
Δ_iter._ (%)	2.439	−0.520	33.339	Δ_iter._ (%)	−21.696	−5.296	−49.446
Δ_NLSM_ (%)	1.526	−1.990	2.806	Δ_NLSM_ (%)	−22.733	−0.137	43.437

**Table 3:** Reconstructed sea state parameters (DG4).

(a) Short Time Duration–600 s				(b) Long Time Duration–3600 s			
Method/Difference	Hs	Tm	γ	Method/Difference	Hs	Tm	γ
	[m]	[s]	[—]		[m]	[s]	[—]
*Input data*				*Input*			
JONSWAP	2.000	5.000	2.500	JONSWAP	2.000	5.000	2.500
*Welch method*				*Welch method*			
Iterative	1.876	4.684	1.491	Iterative	2.031	4.997	2.439
NLSM	1.851	4.789	1.262	NLSM	2.009	5.001	2.466
Δ_iter._ (%)	−6.207	−6.314	−40.364	Δ_iter._ (%)	1.569	−0.061	−2.420
Δ_NLSM_ (%)	−7.456	−4.222	−49.535	Δ_NLSM_ (%)	0.436	0.030	−1.359
*Thomson method*				*Thomson method*			
Iterative	1.863	4.766	1.370	Iterative	2.026	4.989	2.444
NLSM	1.837	4.799	1.268	NLSM	2.003	4.986	2.403
Δ_iter._ (%)	−6.865	−4.678	−45.214	Δ_iter._ (%)	1.298	−0.215	−2.241
Δ_NLSM_ (%)	−8.138	−4.016	−49.269	Δ_NLSM_ (%)	0.169	−0.271	−3.892
*ARMA method*				*ARMA method*			
Iterative	1.702	5.115	1.099	Iterative	1.887	4.872	3.006
NLSM	1.677	5.191	2.415	NLSM	1.868	4.808	2.078
Δ_iter._ (%)	−14.918	2.305	−56.050	Δ_iter._ (%)	−5.653	−2.560	20.230
Δ_NLSM_ (%)	−16.154	3.824	−3.407	Δ_NLSM_ (%)	−6.616	−3.836	−16.866

**Table 4:** Reconstructed sea state parameters (DG5).

(a) Short Time Duration—600 s				(b) Long Time Duration—3600 s			
Method/Difference	Hs	Tm	γ	Method/Difference	Hs	Tm	γ
	[m]	[s]	[—]		[m]	[s]	[—]
*Input data*				*Input*			
JONSWAP	3.000	6.000	1.500	JONSWAP	3.000	6.000	1.500
*Welch method*				*Welch method*			
Iterative	3.088	6.002	1.568	Iterative	3.018	5.920	1.309
NLSM	3.048	6.098	1.859	NLSM	2.976	5.940	1.391
Δ_iter._ (%)	2.948	0.041	4.501	Δ_iter._ (%)	0.594	−1.335	−12.709
Δ_NLSM_ (%)	1.598	1.629	23.95	Δ_NLSM_ (%)	−0.799	−1.002	−7.280
*Thomson method*				*Thomson method*			
Iterative	3.058	6.005	1.251	Iterative	3.031	5.945	1.404
NLSM	3.015	6.082	1.618	NLSM	2.990	5.964	1.406
Δ_iter._ (%)	1.938	0.078	−16.624	Δ_iter._ (%)	1.035	−0.916	−6.395
Δ_NLSM_ (%)	0.507	1.362	7.875	Δ_NLSM_ (%)	−0.335	−0.604	−6.253
*ARMA method*				*ARMA method*			
Iterative	2.313	6.377	1.836	Iterative	2.404	6.291	2.102
NLSM	2.284	6.461	2.606	NLSM	2.375	6.349	2.731
Δ_iter._ (%)	−22.902	6.279	22.370	Δ_iter._ (%)	−19.872	4.858	40.153
Δ_NLSM_ (%)	−23.860	7.680	73.754	Δ_NLSM_ (%)	−20.819	5.813	82.040

**Table 5:** Reconstructed sea state parameters (DG6).

(a) Short Time Duration—600 s				(b) Long Time Duration—3600 s			
Method/Difference	Hs	Tm	γ	Method/Difference	Hs	Tm	γ
	[m]	[s]	[—]		[m]	[s]	[—]
*Input data*				*Input*			
JONSWAP	5.000	9.000	1.000	JONSWAP	5.000	9.000	1.000
*Welch method*				*Welch method*			
Iterative	4.785	8.019	1.000	Iterative	5.091	8.877	1.023
NLSM	4.712	8.711	1.000	NLSM	5.015	8.963	1.000
Δ_iter._ (%)	−4.297	−10.905	0.000	Δ_iter._ (%)	1.814	−1.363	2.320
Δ_NLSM_ (%)	−5.755	−3.211	0.000	Δ_NLSM_ (%)	0.309	−0.406	0.000
*Thomson method*				*Thomson method*			
Iterative	4.829	8.281	1.000	Iterative	5.094	8.995	1.000
NLSM	4.756	8.781	1.000	NLSM	5.017	8.995	1.000
Δ_iter._ (%)	−3.416	−7.993	0.000	Δ_iter._ (%)	1.876	−0.051	0.000
Δ_NLSM_ (%)	−4.887	−2.429	0.000	Δ_NLSM_ (%)	0.349	−0.058	0.000
*ARMA method*				*ARMA method*			
Iterative	3.206	9.560	1.574	Iterative	3.848	9.546	3.443
NLSM	3.164	9.720	2.535	NLSM	3.811	9.479	3.237
Δ_iter._ (%)	−35.882	6.223	57.404	Δ_iter._ (%)	−23.046	6.065	244.252
Δ_NLSM_ (%)	−36.721	7.997	153.467	Δ_NLSM_ (%)	−23.784	5.320	223.686

## References

[1] Piscopo V., Scamardella A. (2014). Passenger ship seakeeping optimization by the Overall Motion Sickness Incidence. Ocean Eng..

[2] Mansard E.P.D., Funke E.R. On the fitting of JONSWP spectra to measured sea states. Proceedings of the 22nd Conference on Coastal Engineering.

[3] Rossi G.B., Crenna F., Piscopo V., Scamardella A. Data processing for the accurate evaluation of sea-waves parameters. Proceedings of the IMEKO TC 19 International Workshop on Metrology for the Sea.

[4] Mansard E.P.D., Funke E.R. On the fitting of parametric models to measured wave spectra. Proceedings of the 2nd International Symposium on Waves and Coastal Engineering.

[5] Det Norske Veritas (2010). Environmental Conditions and Environmental Loads.

[6] Marple S.L. (1987). Digital Spectral Analysis.

[7] Percival D.B., Walden A.T. (1993). Spectral Analysis for Physical Applications.

[8] Schuster A. (1898). On the Investigation of Hidden Periodicities with Application to a Supposed 26 Day Period of Meteorological Phenomena. Terr. Magn..

[9] Welch P.D. (1967). The Use of Fast Fourier Transform for the Estimation of Power Spectra: A Method Based on Time Averaging over Short Modified Periodograms. IEEE Trans. Audio Electroacoust..

[10] Thomson D.J. (1982). Spectrum Estimation and Harmonic Analysis. Proc. IEEE.

[11] Read W.W. (1986). Time Series Analysis of Wave Records and the Search for Wave Groups. Ph.D. Thesis.

[12] Battjes J.A., van Vledder G. Verification of Kimura’s Theory for Wave Group Statistics. Proceedings of the 19th International Conference on Coastal Engineering.

[13] Coleman T.F., Li Y. (1996). Trust Region Approach for Nonlinear Minimization Subject to Bounds. SIAM J. Optim..

[14] Walden A.T., McCoy E.J., Percival D.B. (1995). The effective bandwidth of a multitaper spectral estimator. Biometrika.

[15] Walden A.T., McCoy E.J., Percival D.B. (1994). The Variance of Multitaper Spectrum Estimates for Real Gaussian Processes. IEEE Trans. Signal Process..

[16] Holm S., Hovem J.M. (1979). Estimation of scalar ocean wave spectra by the Maximum Entropy method. IEEE J. Ocean. Eng..

[17] Mandal S., Witz J.A., Lyons G.Y. (1992). Reduced order ARMA spectral estimation of ocean waves. Appl. Ocean Res..

